# Misinformation, knowledge and COVID-19 vaccine acceptance: a cross-sectional study among health care workers and the general population in Kampala, Uganda

**DOI:** 10.1186/s12889-024-17678-9

**Published:** 2024-01-17

**Authors:** Maxine Atuheirwe, Richard Otim, Keneth Junior Male, Stella Ahimbisibwe, Joachim Dzidzor Sackey, Obondo James Sande

**Affiliations:** 1 Department of Immunology and Molecular Biology, School of Biomedical Sciences, College of Health Sciences, Makerere University, P.O.Box 7072, Kampala, Uganda; 2 Department of Biochemistry, Mbarara University of Science and Technology, Mbarara, Uganda; 3 Department of Food Technology and Nutrition, School of Food Technology, Nutrition and Bioengineering (SFTNB), Makerere University, Kampala, Uganda; 4 Department of Urban-Global Public Health, Rutgers School of Public Health, Rutgers Biomedical and Health Sciences, Newark, NJ USA

**Keywords:** COVID-19, COVID-19 vaccine, Misinformation, Health care workers, General population

## Abstract

COVID-19 has greatly affected communities worldwide, more so in low- and middle-income countries. To successfully resolve the COVID-19 pandemic, vaccination coverage of more than 80% is required. However, misinformation has affected this by increasing COVID-19 vaccine hesitancy. Limited studies have assessed the effect of COVID-19 misinformation on vaccine acceptance, especially in Africa. This study assessed people’s knowledge of the COVID-19 vaccine and the effect of misinformation on vaccine uptake among healthcare workers (HCWs) versus the general population in Uganda.

**Methods **This was a cross-sectional quantitative study conducted from January 2022 to June 2022, and involved healthcare workers (HCWs) and the general population of Kampala, Uganda. A structured questionnaire was used to collect data. We recruited 564 study participants, including 311 healthcare workers (HCWs) and 253 from the general population. Data were analyzed using frequency distributions and Chi-square tests. SPSS version 22.0 was used to conduct all study analyses.

**Results **This study revealed that the proportion of vaccinated HCWs (77.4%) was significantly higher than that of the vaccinated general population (64.4%, *p* = 0.010). Nearly all study participants were aware of COVID-19 vaccines (96.7%). The research revealed that a large proportion of the participants (89.7%) encountered rumors regarding unverified adverse effects of the COVID-19 vaccine. This information significantly contributed to vaccine hesitancy, with 81.1% expressing reluctance to receive the vaccine, and 55% stating their unwillingness to get vaccinated. Misinformation affected people’s vaccine acceptance, affecting their willingness to receive vaccines if unvaccinated and potentially influencing their receptiveness to future vaccines or boosters if already vaccinated.

**Conclusions **The study showed a negative impact of misinformation on vaccine uptake and could be the most significant contributor to vaccine hesitancy in future vaccine programs.

## Introduction

On March 11, 2020, the World Health Organization (WHO) declared an outbreak of a new fast-spreading disease, COVID-19 [1]. COVID-19 is caused by severe acute respiratory syndrome coronavirus-2 (SARS-CoV-2) [2]. Currently, the World Health Organization (WHO) has confirmed 772,138,818 cases of COVID-19 worldwide, with 9,556,262 cases reported in Africa. Among them, Uganda has contributed 170,255 identified cases (data from the WHO COVID-19 dashboard and the Ministry of Health, Uganda as of 6th December 2023). Much as Africa has had fewer COVID-19 cases, the WHO warned of consequences likely to result from community transmissions in low-income countries with weak health systems [3]. Indeed, community transmissions have already occurred in Africa [4].

To mitigate community transmissions, several vaccines were approved for use after being tested and proven safe to generate an effective immune response [5, 6]. However, the effectiveness of vaccines in achieving herd immunity significantly relies on being accepted by at least 55% of the population [7]. Given the well-documented evidence of vaccine hesitancy worldwide, often influenced by online and offline misinformation, achieving herd immunity may be challenging [7, 8].

Research on the impact of misinformation on COVID-19 vaccine acceptance within U.S. and UK populations has revealed that misinformation led to a 6.4% decrease in vaccine uptake in the UK and a 2.4% decline in the US [9]. Studies carried out in June 2020 revealed that the projected acceptance rates of the COVID-19 vaccine were 38% and 34.3% among the surveyed populations in the UK and the U.S., respectively [8, 10, 11]. A study among Chinese healthcare workers (HCWs) showed that a larger percentage of health personnel were open to receiving the vaccine compared to the general population [12].

A study conducted in Uganda among high-risk populations revealed that 70.1% expressed willingness to accept the COVID-19 vaccine, with the odds of acceptance being four times higher in males than females [13]. Another investigation involving medical students in Uganda disclosed a vaccine acceptance rate of 37.3%, with a higher likelihood of acceptance observed among male and single participants [14]. Research conducted among the general population in western Uganda also showed that males were more likely to accept a vaccine than females [15]. Research conducted among Ugandan eye healthcare workers revealed that 97.6% of the participants had accepted or expressed willingness to take the COVID-19 vaccine [16]. Given the scarcity of information regarding the influence of misinformation on vaccine uptake and the public's knowledge about COVID-19 vaccines, it is crucial to investigate the level of knowledge among the general population and healthcare workers (HCWs) in Uganda. Additionally, exploring the impact of prevalent misinformation on COVID-19 vaccine acceptance is imperative. This study aimed to highlight this gap.

## Materials and methods

### Study design

This was a quantitative cross-sectional study that evaluated the knowledge and acceptance of the COVID-19 vaccine and the impact of misinformation on the intent of getting vaccinated. The study targeted healthcare workers (HCWs) and the general population in Uganda and was conducted from January 2022 to June 2022.

### Study site and setting

The study was conducted in the Kampala metropolitan area, Uganda. Demographic data and data associated with COVID-19 uptake were collected from HCWs and the general population living/ working in various places in the Kampala metropolitan area, Uganda. The various study sites included Mulago Hospital, Makerere University, Wandegeya, Kikoni, National Water and Sewerage Corporation Gabba, the National Water and Sewerage Corporation Kampala headquarters, Kibuli Hospital, International Resource Center Kampala Bugoloobi and Islamic University in Uganda.

### Study participants

This study recruited HCWs and individuals in the general population aged 18 and above living in Kampala, Uganda.

### Sample size estimation

To address the study objectives, we calculated the sample size using the formula for proportion studies, according to Kevin M. Sullivan accessible at OpenEpi (https://www.openepi.com/SampleSize/SSPropor.htm ) Version 3 [17].

$$\mathrm{Sample size}\mathrm{n }= \left[{\text{DEFF}}*{\text{Np}}\left(1-{\text{p}}\right)\right]/ \left[\left({\text{d}}2/{\text{Z}}21-\mathrm{\alpha }/2*\left({\text{N}}-1\right)\right)\right]+{\text{p}}*\left(1-{\text{p}}\right)]$$

*where, n* = sample size, DEFF = Design effect, = 1 *N* = Population size, 1659600 *p* = Proportion of individuals who will accept the vaccine in Uganda is estimated to be 37.3% from a study in Uganda [14] *d* = desired absolute precision or absolute level of precision, = 5% Z = 1.96

Substituting the above estimates into the formula, different sample sizes were obtained with varying confidence levels, as shown below. Based on these results, we deemed a sample size of 564 adequate to ensure a sufficient level of statistical power.

### Sample size for frequency in a population

**Table:** 

Confidence Level (%)	Sample Size
80%	360
90%	154
95%	254
97%	441
99%	620
99.9%	1013

### Participant selection criteria

We recruited HCWs and individuals from the public aged 18 and above living and working in the Kampala metropolitan area between January 2022 and June 2022. We randomly recruited both females and males in the study to minimize bias.

### Ethical approval

Ethical approval to conduct the study was obtained from the School of Biomedical Sciences Research and Ethics Committee, protocol No. SBS-2021–76 and the Uganda National Council of Science and Technology (HS2013ES). The Ministry of Health provided administrative clearances for conducting the study in the different healthcare institutions in Kampala, Uganda. After participant recruitment into the study, informed consent was obtained.

### Data collection

This was a quantitative cross-sectional study conducted between January 2022 and June 2022. A structured questionnaire consisting of 5 major sections was used to collect data: Demographic information, knowledge, beliefs, and attitudes about coronavirus, COVID-19 vaccine, and misinformation. A previously designed questionnaire was adopted and modified [8, 18]. The questions were first pre-tested, revised, and finalized based on feedback from testers. The study targeted HCWs and the general population living in Kampala, Uganda. Participants were presented with questionnaires to fill out physically. Each participant received a questionnaire for completion, and in cases where individuals were not literate, a data collector assisted in filling out the questionnaire on their behalf. Of the 564 participants, 311 were HCWs, and 253 were the general population.

### Data analysis

Data were extracted from questionnaires into SPSS version 22.0, where all analyses were done. Chi-square tests were used for all analyses as presented in all the tables to enable a comparison of the COVID-19 vaccine knowledge and the impact of misinformation on the intent to get vaccinated between the HCWs and the general population. The study incorporated five knowledge-based questions to evaluate participants' knowledge levels. This assessment relied on participants' responses to the knowledge questions, categorizing correct responses as "Yes" or "Strongly Agree" and incorrect responses as "No" or "Strongly Disagree." Responses like "Tend to Agree" and "Tend to Disagree" were considered uncertain. The significance threshold was established at a *p*-value of less than 0.05. Research findings were organized and presented using percentages and graphical representations. The study presented the demographic characteristics of participants, their existing knowledge of COVID-19 and COVID-19 vaccination, and the sharing of COVID-19 vaccine information. It's crucial to note that the study did not undertake a sensitivity analysis.

## Results

### The general profile of study participants

The study enrolled 564 participants living and/or working in Kampala, Uganda. The study populations were HCWs (people working in a health care setting such as physicians, nurses, technicians, technologists, physiotherapists, optometrists, supervisors of health care services and personal care, medical interns, biomedical engineers, pharmacists, dentists, occupational therapists, midwives, cleaners, receptionists among others) and general population members were participants not working in a health care entity or not directly involved in health care service delivery.

This study showed that both gender (*p*= 0.0017) and age (*p*<0.001) categories were significantly different among the study groups. More than half of the population were males (55.7%), and most of the participants were aged between 18-30 years (72.7%). The study uncovered significant disparities in education and income levels among the study populations. These discrepancies were starkly evident with education (*p*<0.001) and income (*p*=0.018). A noteworthy 43.5% of participants had attained a bachelor's degree, while 43.3% were engaged in full-time employment. In addition, a substantial 61.9% of the study population earned more than 500,000 Uganda shillings monthly. Another intriguing facet of our findings was the composition of households. Most participants lived in households inhabited by 3 to 4 individuals, accounting for 32.3% of the surveyed population.

The most captivating revelation of our study was the vaccination status of the participants. An impressive 71.6% of the study cohort had been vaccinated. Strikingly, healthcare workers (HCWs) demonstrated a significantly higher vaccination rate, with 77.4% of them having received the vaccine, compared to 64.4% of the general population (*p*=0.010). This divergence in vaccination rates adds an interesting layer to our understanding of healthcare practices and underscores the need for further investigation and intervention (Table 1).

**Table 1:** Shows the general profile of the study participants

General profile	General population, n (%)	Healthcare workers n (%)	*P* value
**Age, *N* = 564**			
18–30	168 (64.4%)	265 (84.6%)	** < 0.001**
31–45	67 (26.5%)	42 (13.5%)	
46–60	17 (6.7%)	4 (1.3%)	
Above 60	1 (0.4%)	0 (0.0%)	
**Gender, *N* = 564**			
Female	98 (38.7%)	152 (48.9%)	**0.017**
Male	155 (61.3%)	159 (51.1%)	
**Level of education, *N* = 557**			
Primary	4 (1.6%)	1 (0.3%)	** < 0.001**
O level	17 (6.9%)	7 (2.3%)	
A level	41 (16.5%)	22 (7.1%)	
Certificate	15 (6.0%)	44 (14.2%)	
Diploma	20 (8.1%)	91 (29.4%)	
Bachelor’s Degree	118 (47.6%)	125 (40.5%)	
Post graduate degree	32 (12.9%)	18 (5.8%)	
Other technical qualification	1 (0.4%)	1 (0.3%)	
**Employment status, *N* = 545**			
Full time	114 (47.5%)	122 (40.0%)	
Part-time	51 (21.3%)	125 (41.0%)	
Unemployed	37 (15.4%)	10 (3.3%)	
Student	35 (14.6%)	44 (14.4%)	
Other	3 (1.2%)	4 (1.3%)	
**Gross income, *N* = 423**			
Under 500,000	47 (26.9%)	63 (25.4%)	**0.018**
500,000–1,000,000	54 (30.9%)	98 (39.5%)	
1,000,000–5,000,000	41 (23.4%)	55 (22.2%)	
5,000,000 or over	8 (4.6%)	18 (7.3%)	
Don’t know	25 (14.3%)	14 (5.6%)	
**Household members, *N* = 564**			
1	24 (10.3%)	22 (7.4%)	0.676
2	33 (14.1%)	46 (15.5%)	
3–4	70 (29.9%)	101 (34.1%)	
5–6	67 (28.6%)	79 (26.7%)	
7 or more	40 (17.1%)	48 (16.2%)	
**Vaccination status, *N* = 563**			**0.010**
Vaccinated	163 (64.4%)	240 (77.4%)	
Not vaccinated	63 (24.9%)	51 (16.5%)	
Plan to get vaccinated	15 (5.9%)	12 (3.9%)	
Don’t intend to get vaccinated	8 (3.2%)	6 (1.9%)	
Unsure about vaccination plans	4 (1.6%)	1 (0.3%)	

*N* total participants, *n* number of respondents, Chi-square test was used to obtain *p* values

### Participants’ knowledge of COVID-19 vaccines

Remarkably, the vast majority of the study's participants, amounting to 96.7%, were aware of the existence of COVID-19 vaccines. This high awareness level emphasizes the global prominence and widespread recognition of these vaccines. Moreover, over two-thirds of the participants, precisely 68.7%, displayed awareness of the wide availability of these vaccines. A significant number of participants (50.4%) strongly agreed that vaccines were essential in controlling the spread of the disease, with 45.3% of the participants firmly believing in the effectiveness of these vaccines against COVID-19. Safety, a paramount concern for many, was also a subject of firm agreement, with 46.8% expressing their conviction in the safety of these vaccines. One particularly noteworthy finding was the significant number of participants who possessed knowledge about the widespread availability of COVID-19 vaccines. A *p*-value of 0.013 validates this, signifying the statistical significance of this knowledge among the participants (Table 2).

**Table 2:** Shows participants' knowledge of COVID-19 vaccination

Knowledge	General population, n (%)	HCWs n (%)	*P* value
**COVID-19 vaccine awareness, *N* = 511**			
Yes	219 (95.2%)	275 (97.9%)	0.136
No	11 (4.8%)	6 (2.1%)	
**Is there a widely available vaccine, *N* = 534**			
Yes	152 (63.6%)	215 (72.9%)	**0.013**
No	44 (18.4%)	51 (17.3%)	
Don’t know	43 (18%)	28 (9.5%)	
Prefer not to say	0 (%)	1 (0.3%)	
**Overall, vaccines are important, *N* = 547**			
Strongly agree	123 (50%)	153 (50.8%)	0.531
Tend to agree	47 (19.1%)	59 (19.6%)	
Tend to disagree	21(8.5%)	36 (12.0%)	
Strongly disagree	36 (14.6%)	36 (12.0%)	
Don’t know	19 (7.7%)	17 (5.6%)	
**Overall, vaccines are effective, *N* = 541**			
Strongly agree	110 (45.3%)	135 (45.3%)	0.301
Tend to agree	81 (33.3%)	100 (33.6%)	
Tend to disagree	25 (10.3%)	30 (10.1%)	
Strongly disagree	13 (5.3%)	25 (8.4%)	
Don’t know	14 (5.8%)	8 (2.7%)	
**Overall, vaccines are safe, *N* = 549**			
Strongly agree	107 (43.3%)	146 (48.3%)	0.297
Tend to agree	86 (34.8%)	102 (33.8%)	
Tend to disagree	23 (9.3%)	20 (6.6%)	
Strongly disagree	20 (8.1%)	28 (9.3%)	
Don’t know	11 (4.5%)	6 (2.0%)	

*N* total participants, *n* number of respondents, The Chi-square test was used to obtain *p*-values

### Use of social media platforms and information sharing on the COVID-19 vaccine

This study provided a fascinating glimpse into the diverse and widespread use of social media platforms among the participants, shedding light on their digital engagement habits and media consumption patterns.

The study revealed that a substantial portion, specifically 29.3% of the participants, confined their social media usage to a maximum of one platform. Among these platforms, WhatsApp stood out as the most common singly used platform of choice, with an impressive 68.5% opting for this widely popular communication tool. On the other end of the spectrum, a small minority, totaling 3.9%, abstained from using any of the social media platforms altogether. An even more diminutive fraction, just 0.4%, ventured into the realm of other social media platforms not included in the study's designated list.

An interesting finding was that a notable portion of the participants, constituting 8%, displayed digital versatility by actively engaging with at least two distinct social media platforms in their daily routines. This diversity was apparent in the finding that over 44.4% of the participants integrated both WhatsApp and Facebook into their online interactions. Taking a broader perspective, it was apparent that over 58.4% of the participants were engaged with more than two social media platforms. This study revealed that a significant number of participants, totaling 37.6%, dedicated more than 3 h of their daily lives to social media, while a mere 4.3% chose to abstain entirely from social media. The study also extended its inquiry to the participants' sources of information consumption. Intriguingly, 4.4% of the participants derived information from all the media platforms covered in the study. Television (TV) stood out as the primary medium of choice for information consumption, with 15.6% of the participants relying solely on this traditional source for their informational needs (Table 3).

**Table 3:** Shows the social media platforms used and receiving information on the COVID-19 vaccine

Platforms used	Frequency	Total, N	Percentage (%)
Facebook	26	563	4.6%
twitter	15		2.7%
You tube	4		0.7%
WhatsApp	113		20.1%
Instagram	5		0.9%
Pinterest	1		0.2%
LinkedIn	1		0.2%
other	2		0.4%
None of the above	22		3.9%
Facebook, twitter	1		0.2%
Facebook, WhatsApp	20		3.6%
Twitter, WhatsApp	5		0.9%
Twitter, LinkedIn	2		0.4%
YouTube, WhatsApp	11		2.0%
WhatsApp, Instagram	3		0.5%
WhatsApp, LinkedIn	3		0.5%
More than 2 combinations	329		58.4%
**Time spent on social media**			
none	24	559	4.3%
Below 1 h	161		29.3%
1 h 3hoours	164		29.3%
More than 3 h a day	210		37.6%
**Receiving information on COVID-19**			
Television news	88	563	15.6%
Radio, podcasts and other broadcasts	11		2.0%
Newspapers and other journalism	5		0.9%
Daily government briefings	5		0.9%
International health authorities i.e., WHO	10		1.8%
Healthcare workers	7		1.2%
Scientific experts	3		0.5%
Government websites	1		0.2%
Social media platforms e.g., Facebook,twitter, you tube	19		3.4%
More than 1 combinations	414		73.5%

*N* total participants, *n* number of respondents, The Chi-square test was used to obtain *p*-values

### Exposure to misinformation (‘rumors’)

This study explored the concerning impact of COVID-19 vaccine-related rumors on participants' intent to get vaccinated. A significant number of participants (89.7%) were exposed to unverified rumors, which had a profound effect on them. These rumors caused fear of getting vaccinated among the participants (81.1%) while 55% expressed reluctance to get vaccinated, indicating that misinformation was a significant barrier to vaccination.

The study showed that before exposure to misinformation, the majority (64.2%) were willing to get vaccinated, suggesting that their initial attitudes toward vaccination were positive. However, nearly half of the participants (46.9%) believed these rumors to be true or somewhat true, highlighting the effectiveness of misinformation in instilling doubt and fear.

Additionally, a substantial 71.4% of study participants were inclined to share these rumors with their social circles, highlighting the potential for false information to spread further. This situation underscores the urgent need for effective vaccine education and communication to combat the influence of misinformation and promote accurate vaccine-related knowledge (Table 4).

**Table 4:** **S**hows exposure to Misinformation (‘rumors’) and its effect on intent to get vaccinated

Misinformation	General population, n (%)	HCWs n (%)	*P* value
**Have you been exposed to rumors circulating about bad effects of COVID-19 vaccine, *N* = 542**			
Yes	211 (87.2%)	275 (91.7%)	0.118
No	31 (12.8%)	25 (8.3%)	
**Have you heard information that scared you about taking the vaccine, *N* = 528**			
Yes	182 (78.8%)	246 (82.8%)	0.263
No	49 (21.2%)	51 (17.2%)	
**Did exposure to such information affect your intent to get vaccinated, *N* = 489**			
Yes definitely	120 (56.1%)	149 (54.2%)	0.488
No	86 (40.2%)	109 (39.6%)	
Not sure	8 (3.7%)	17 (6.2%)	
**Before getting exposed to such information, were you willing to get vaccinated, *N* = 491**			
Yes definitely	143 (67.5%)	172 (61.6%)	0.180
no	51 (24.1%)	69 (24.7%)	
Not sure	18 (8.5%)	38 (13.6%)	
**Overall, how much do you agree with the rumors you have heard about the effects of COVID-19 vaccine, *N* = 516**			
Strongly agree	49 (21.4%)	55(19.2%)	0.702
Somewhat agree	63 (27.5%)	75 (26.1%)	
Neither agree nor disagree	47 (20.5%)	53 (18.5%)	
Somewhat disagree	28 (12.2%)	40 (13.9%)	
Strongly disagree	29 (12.7%)	50 (17.4%)	
Don’t know	13 (5.7%)	14 (4.9%)	
**Overall, how likely are you to share this information with your friends and followers, *N* = 547**			
Very likely	127 (51.2%)	152 (50.8%)	0.364
Somewhat likely	53 (21.4%)	59 (19.7%)	
Neither likely nor unlikely	17 (6.9%)	23 (7.7%)	
Somewhat unlikely	8 (3.2%)	18 (6.0%)	
Very unlikely	25 (10.1%)	35 (11.7%)	
Don’t know	18 (7.3%)	12 (4.0%)	

*N* total participants, *n* number of respondents, Chi-square test was used to obtain *p* values

### Fears about COVID-19 vaccination

The study also delved into participants' diverse concerns about COVID-19 vaccines. Much as most of the participants strongly expressed worry about experiencing bad side effects from the COVID-19 vaccines (60.4%), a good majority did not expect to acquire COVID-19 from the vaccines (63.7%). In contrast, most participants believed that COVID-19 vaccines were too new to be trusted (49.1%).

The highest percentage of participants believed to have adequate knowledge about the COVID-19 vaccine to make an informed decision to get vaccinated (46.4%), and the difference in knowledge about the vaccines was statistically significant between the two study groups (*p* = 0.001). The study revealed a strong inclination to follow government (45.3%) and healthcare professional (53.1%) recommendations for vaccination, highlighting the influence of authoritative endorsements.

Overall, most of the participants strongly believed that vaccines were compatible with their religion (53.2%). Furthermore, over 57.8% of participants believed that even after vaccination, it was essential to maintain social distancing and preventive measures, recognizing vaccines as part of a broader pandemic-fighting strategy (Table 5).

**Table 5:** Shows participant’s fears about the COVID-19 vaccine

Fears	General population, n (%)	HCWs n (%)	*P* value
**I would be worried about experiencing side effects from COVID-19 vaccination, *N* = 541**			
Strongly disagree	36 (15.1%)	34 (11.3%)	0.551
Tend to disagree	8 (3.3%)	10 (3.3%)	
Don’t know	14 (5.9%)	26 (8.6%)	
Tend to agree	36 (15.1%)	50 (16.6%)	
Strongly agree	145 (60.7%)	182 (60.3%)	
**A COVID-19 vaccine could give me COVID-19, *N* = 542**			
Strongly disagree	153 (63.7%)	192 (63.6%)	0.824
Tend to disagree	19 (7.9%)	20 (6.6%)	
Don’t know	15 (6.2%)	17 (5.6%)	
Tend to agree	14 (5.8%)	25 (8.3%)	
Strongly agree	39 (16.2%)	48 (15.9%)	
**COVID-19 vaccines will be too new for me to be confident about getting vaccinated, *N* = 534**			
Strongly disagree	53 (22.4%)	76 (25.6%)	0.394
Tend to disagree	17 (7.1%)	21 (7.1%)	
Don’t know	12 (5.1%)	26 (8.8%)	
Tend to agree	31 (13.1%)	36 (12.1%)	
Strongly agree	124 (52.3%)	138 (46.5%)	
**I know enough about COVID -19 vaccine to make an informed decision on whether to get vaccinated, *N* = 541**			
Strongly disagree	76 (31.4%)	57 (19.1%)	**0.001**
Tend to disagree	17 (7.0%)	10 (3.3%)	
Don’t know	18 (7.4%)	21 (7.0%)	
Tend to agree	35 (14.5%)	56 (18.7%)	
Strongly agree	96 (39.7%)	155 (51.8%)	
**Overall, vaccines are compatible with my religious beliefs, N = 549**			
Strongly agree	121 (49.0%)	171 (56.6%)	
Tend to agree	37 (15.0%)	56 (18.5%)	
Tend to disagree	19 (7.7%)	26 (8.6%)	
Strongly disagree	45(18.2%)	37 (12.3%)	
Don’t know	25 (10.1%)	12 (4.0%)	

*N* total participants, *n* number of respondents, Chi-square test was used to obtain *p* values

## Discussion

We present here our study findings of people’s knowledge and the effect of misinformation on COVID-19 vaccine uptake among HCWs versus the general population in Uganda. It is reassuring to note that a substantial number of participants were vaccinated (71.6%). Among the vaccinated individuals, the proportion of HCWs (77.4%) was significantly higher than the proportion of the general population (64.4%). These results are comparable to results from a study conducted in China that indicated that 76.98% of healthcare workers accepted the COVID-19 vaccine, compared to 56.19% of the vaccinated general population [19]. The lower rates of vaccine hesitancy in HCWs than in the general population could be attributed to adequate knowledge about COVID-19 among HCWs.

According to the study results, 96.7% of the respondents demonstrated awareness of the presence of COVID-19 vaccines. This high level of awareness may have contributed to a more informed and receptive attitude towards vaccination initiatives. These study results are consistent with studies conducted elsewhere that showed the study populations were knowledgeable about COVID-19 and COVID-19 vaccines [20–22].

This study indicated that fewer HCWs stayed at home whilst they were vulnerable compared to the general population, possibly because most HCWs were frontline workers during the pandemic and continued working during lockdowns, unlike most of the general population. Much as our study indicated a significantly higher number of HCWs knew of widely available vaccines than the general population, both study groups were equally aware of COVID-19 preventive procedures (such as hand washing, wearing a mask, and reducing contact with others). This is comparable to results obtained from research conducted in Saudi Arabia, indicating that about 90% of participants were knowledgeable about COVID-19 preventive procedures [23].

Over 96% of the study participants used social platforms, with WhatsApp and Facebook being the most used platforms. These results are similar to those obtained in other studies done elsewhere where WhatsApp and Facebook were the most commonly utilized platforms [24, 25]. Also, the study showed that participants received COVID-19-related information from more than one media platform with television in combination with other media platforms being used the most. This is in agreement with results from a study in India that showed television was among the most commonly used platforms [26]. Therefore, to reach a greater percentage of Ugandans in urban and peri-urban areas, about COVID-19 and its preventive measures, different channels of communication, such as TV and other social media platforms, such as Facebook and WhatsApp, should be utilized.

The study indicated no difference in the exposure to misinformation between the study groups. Before exposure to misinformation, most of the participants were prepared to take up the vaccine, though this percentage significantly reduced after exposure to the misinformation. These results significantly align with results from a study done previously among populations from the UK and the US where intent to vaccinate decreased by 6.4% and 2.4%, respectively [27]. This implies that wrong information about vaccines negatively affects vaccination programs. Much as most participants were more likely to fact-check the rumors via other sources, there was no statistical difference in the number of HCWs versus the general population who were likely to confirm the rumors.

The study showed both study groups were equally worried about experiencing side effects from the COVID-19 vaccine, and also believed that the COVID-19 vaccine could give them COVID-19. Also, both study groups similarly believed that COVID-19 vaccines would be too new for them to be confident about getting vaccinated. This is because the COVID-19 vaccine was possibly new and there was limited data about COVID-19 side effects.

Much as the study showed more HCWs believed to know enough about the COVID-19 vaccine to make an informed decision on whether to get vaccinated compared to the general population, both study groups equally believed they would get vaccinated if the government and a health care professional recommended a COVID-19 vaccine. These results match another study showing that the health sector, government, scientists, and politicians were essential during the COVID-19 pandemic [28]. This implies that, in efforts to encourage COVID-19 vaccine uptake, both government officials and HCWs are equally important.

Both study groups equally believed that if they were vaccinated, they still had to follow social distancing and other restrictions. This is in line with study results that showed both study groups were knowledgeable about COVID-19 and the COVID-19 vaccine and had maximum compliance with COVID-19 guidelines. This also indicates that participants understood that vaccination doesn’t protect one from catching COVID-19 but protects against COVID-19 severe forms.

## Strengths and limitations

### Limitations

Many participants were worried that disclosing their vaccination status might have negative consequences for their employment, including the possibility of job termination. There was also apprehension that government authorities might actively seek out the unvaccinated, causing hesitation in participating due to concerns about repercussions related to their vaccination status becoming disclosed. Additionally, the study focused on adult respondents, excluding those under 18 years, who are known to frequently use social media for information. Future research should consider including younger participants to gain insights into their misinformation exposure and beliefs.

### Strength

Our research, based on questionnaire surveys, efficiently collected data from diverse participant groups while maintaining standardization to reduce biases and enhance data reliability. Participant anonymity, particularly on sensitive topics like vaccine status, was safeguarded. Additionally, this survey method proved cost-effective, enabling large-scale research within budget limitations. It generated objective data by mitigating potential interviewer bias, as participants directly provided their responses.

## Conclusion

Most of the HCWs and the general population recruited in the study were knowledgeable about the coronavirus, COVID-19, and the COVID-19 vaccine. Both groups were also aware of the COVID-19 preventive measures and knew it was necessary to undertake the COVID-19 measures even after vaccination. The study indicated a high trust in government and healthcare workers regarding any information and recommendations about COVID-19 and the COVID-19 vaccine. These are the most common trusted information sources and should be maximumly exploited to increase accurate information spread. The study showed a negative impact of misinformation on vaccine uptake and could be the biggest contributor to vaccine hesitancy. More reliable and accurate information is needed to increase COVID-19 vaccine uptake and booster dose uptake, and its increased dissemination via many platforms is vital. This will enhance information reaching out to many people not only in the urban setup but also in rural regions. Based on the small sample size of 564 and the age range in the study, i.e. 18–30, it may not be right to generalize the results to the Ugandan population. Exploring this in the future countrywide studies can give more generalizable findings.

## Recommendation

Since most participants mainly use Facebook and WhatsApp and also obtain COVID-19-related information from majorly TV and social media, more emphasis should be put on circulating accurate and reliable information via these sources to increase vaccine uptake, especially in the general population. More emphasis should be directed on encouraging vaccinated individuals to take COVID-19 boosters. Also, more research work aimed at understanding the effect of circulating misinformation on vaccine uptake among healthcare workers versus the general population needs to be done in rural setups in Uganda that are given less priority. In addition, the Uganda Communications Commission should devise means of regulating the untruthful information spread about COVID-19 on social media platforms by mostly uninformed persons.

## Data Availability

The datasets used and/or analyzed during the current study are available from the corresponding author upon reasonable request.

## Abbreviations

COVID-19: Coronavirus disease 2019
HCWs: Health Care Workers
WHO: Word Health Organization
SPSS: Statistical Package for the Social Sciences
NIHR: National Institute for Health and Care Research
RSTMH: Royal Society of Tropical Medicine and Hygiene

## References

[1] Guo Y-R, Cao Q-D, Hong Z-S, Tan Y-Y, Chen S-D, Jin H-J (2020). The origin, transmission and clinical therapies on coronavirus disease 2019 (COVID-19) outbreak–an update on the status. Mil Med Res.

[2] Remuzzi A, Remuzzi G (2020). COVID-19 and Italy: what next?. Lancet.

[3] Attaran B (2021). Advice on the use of masks in the context of COVID-19: interim guidance, 5 June 2020. Iranian J Biol.

[4] Ikoona EN, Kitara DL (2021). A proposed framework to limit post-lockdown community transmission of COVID-19 in Africa. The Pan Afr Med J.

[5] Knoll MD, Wonodi C (2021). Oxford–AstraZeneca COVID-19 vaccine efficacy. The Lancet.

[6] Mahase E. Covid-19: AstraZeneca vaccine is not linked to increased risk of blood clots, finds European Medicine Agency. London: British Medical Journal Publishing Group; 2021. 10.1136/bmj.n774 33741638

[7] Khubchandani J, Sharma S, Price JH, Wiblishauser MJ, Sharma M, Webb FJ (2021). COVID-19 vaccination hesitancy in the United States: a rapid national assessment. J Community Health.

[8] Sherman SM, Smith LE, Sim J, Amlôt R, Cutts M, Dasch H (2021). COVID-19 vaccination intention in the UK: results from the COVID-19 vaccination acceptability study (CoVAccS), a nationally representative cross-sectional survey. Hum Vaccin Immunother.

[9] Loomba S, de Figueiredo A, Piatek S, de Graaf K, Larson HJ. Measuring the impact of exposure to COVID-19 vaccine misinformation on vaccine intent in the UK and US. MedRxiv. 2020:2020.10. 22.20217513.

[10] Lazarus JV, Ratzan SC, Palayew A, Gostin LO, Larson HJ, Rabin K (2021). A global survey of potential acceptance of a COVID-19 vaccine. Nat Med.

[11] Malik AA, McFadden SM, Elharake J, Omer SB (2020). Determinants of COVID-19 vaccine acceptance in the US. EClinicalMedicine.

[12] Fu C, Wei Z, Zhu F, Pei S, Li S, Zhang L, et al. Acceptance of and preference for COVID-19 vaccination in healthcare workers: a comparative analysis and discrete choice experiment. MedRxiv. 2020:2020.04. 09.20060103.

[13] Bongomin F, Olum R, Andia-Biraro I, Nakwagala FN, Hassan KH, Nassozi DR (2021). COVID-19 vaccine acceptance among high-risk populations in Uganda. Ther Adv Infect Dis.

[14] Kanyike AM, Olum R, Kajjimu J, Ojilong D, Akech GM, Nassozi DR (2021). Acceptance of the coronavirus disease-2019 vaccine among medical students in Uganda. Trop Med Health.

[15] Echoru I, Ajambo PD (2020). Acceptance and risk perception of COVID-19 vaccine in Uganda: a cross sectional study in Western Uganda.

[16] Otiti-Sengeri J, Andrew OB, Lusobya RC, Atukunda I, Nalukenge C, Kalinaki A (2022). High COVID-19 vaccine acceptance among eye healthcare workers in Uganda. Vaccines.

[17] Alrokban AH, et al. Bullying and its risk factors among elementary school children in Riyadh, Saudi Arabia. Int Res J Public Environ Health. 2019;6:105–14.

[18] Loomba S, Maertens R, Roozenbeek J, Götz FM, van der Linden S, De Figueiredo A. Ability to detect fake news predicts sub-national variation in COVID-19 vaccine uptake across the UK. medRxiv. 2023:2023.05. 10.23289764.

[19] Fu C, Pei S, Li S, Sun X, Liu P. Acceptance and preference for COVID-19 vaccination in health-care workers (HCWs). MedRxiv. 2020.

[20] Qutob N, Awartani F (2021). Knowledge, attitudes and practices (KAP) towards COVID-19 among Palestinians during the COVID-19 outbreak: A cross-sectional survey. PLoS ONE.

[21] Person B, Sy F, Holton K, Govert B, Liang A, Team SCO (2004). Fear and stigma: the epidemic within the SARS outbreak. Emerg Infect Dis.

[22] Rugarabamu S, Ibrahim M, Byanaku A (2020). Knowledge, attitudes, and practices (KAP) towards COVID-19: a quick online cross-sectional survey among Tanzanian residents. MedRxiv.

[23] Bazaid AS, Aldarhami A, Binsaleh NK, Sherwani S, Althomali OW (2020). Knowledge and practice of personal protective measures during the COVID-19 pandemic: A cross-sectional study in Saudi Arabia. PLoS ONE.

[24] Adekoya CO, Fasae JK (2021). Social media and the spread of COVID-19 infodemic. Glob Knowl Mem Commun.

[25] Obi-Ani NA, Anikwenze C, Isiani MC (2020). Social media and the Covid-19 pandemic: Observations from Nigeria. Cogent arts Humanit.

[26] Parikh PA, Shah BV, Phatak AG, Vadnerkar AC, Uttekar S, Thacker N (2020). COVID-19 pandemic: knowledge and perceptions of the public and healthcare professionals. Cureus.

[27] Loomba S, de Figueiredo A, Piatek SJ, de Graaf K, Larson HJ (2021). Measuring the impact of COVID-19 vaccine misinformation on vaccination intent in the UK and USA. Nat Hum Behav.

[28] Sibley CG, Greaves LM, Satherley N, Wilson MS, Overall NC, Lee CH (2020). Effects of the COVID-19 pandemic and nationwide lockdown on trust, attitudes toward government, and well-being. Am Psychol.

